# Metabolomic fingerprint reveals that metformin impairs one-carbon metabolism in a manner similar to the antifolate class of chemotherapy drugs

**DOI:** 10.18632/aging.100472

**Published:** 2012-07-22

**Authors:** Bruna Corominas-Faja, Rosa Quirantes-Piné, Cristina Oliveras-Ferraros, Alejandro Vazquez-Martin, Sílvia Cufí, Begoña Martin-Castillo, Vicente Micol, Jorge Joven, Antonio Segura-Carretero, Javier A. Menendez

**Affiliations:** ^1^ Translational Research Laboratory, Catalan Institute of Oncology, Girona, Spain; ^2^ Girona Biomedical Research Institute, Girona, Spain; ^3^ Department of Analytical Chemistry, Faculty of Sciences, University of Granada, Granada, Spain; ^4^ Research and Development of Functional Food Centre (CIDAF), Health Science Technological Park, Granada, Spain; ^5^ Clinical Research Unit, Catalan Institute of Oncology, Girona, Spain; ^6^ Molecular and Cellular Biology Institute (IBMC), Miguel Hernández University, Elche, Spain; ^7^ Unitat de Recerca Biomèdica (URB-CRB), Institut d'Investigació Sanitària Pere Virgili (IISPV), Universitat Rovira i Virgili, Reus, Catalonia, Spain

**Keywords:** metformin, folic acid, antifolates, AMPK, ATM, pemetrexed, cancer

## Abstract

Metabolomic fingerprint of breast cancer cells treated with the antidiabetic drug metformin revealed a significant accumulation of 5-formimino-tetrahydrofolate, one of the tetrahydrofolate forms carrying activated one-carbon units that are essential for the de novo synthesis of purines and pyrimidines. De novo synthesis of glutathione, a folate-dependent pathway interconnected with one-carbon metabolism was concomitantly depleted in response to metformin. End-product reversal studies demonstrated that thymidine alone leads to a significant but incomplete protection from metformin's cytostatic effects. The addition of the substrate hypoxanthine for the purine salvage pathway produces major rightward shifts in metformin's growth inhibition curves. Metformin treatment failed to activate the DNA repair protein ATM kinase and the metabolic tumor suppressor AMPK when thymidine and hypoxanthine were present in the extracellular milieu. Our current findings suggest for the first time that metformin can function as an antifolate chemotherapeutic agent that induces the ATM/AMPK tumor suppressor axis secondarily following the alteration of the carbon flow through the folate-related one-carbon metabolic pathways.

## INTRODUCTION

The exact site of action and molecular mechanism by which the anti-diabetic, biguanide metformin exerts its recently recognized anticancer activity is poorly understood and remains largely controversial [1-5]. Most of the cell-autonomous mechanisms describing metformin's anticancer activity have been associated with its ability to activate the metabolic rheostat AMP-activated protein kinase (AMPK) [6-10].

Although metformin has been unambiguously demonstrated to activate AMPK by increasing the levels AMP and/or ADP (i.e., AMPK complexes bearing an R531 mutation that renders the AMPK γ2 regulatory complexes insensitive to increases in both ADP and AMP are refractory to metformin's activating effects) [11], the mechanism by which metformin increases intracellular AMP production remains unclear [12]. Here, we accumulate key arguments against the concept that metformin acts *in vivo* as a *“mitochondrial poison”* that inhibits complex I of the respiratory chain, which consequently leads to an imbalance in the AMP/ATP ratio (the extremely sensitive indicator of the intracellular energy status, which is the molecular parameter monitored by AMPK) [13-15]. Beyond the modest extent to which metformin truly interrupts mitochondrial energy formation [16, 17], it is noteworthy that diabetes-associated insulin resistance can be ameliorated by suppressing the oxidative damage that arises from mitochondrial inhibition [18]; therefore, mitochondrial inhibition is an unlikely site of action for an insulin-sensitizing agent like metformin. Perhaps more importantly, metformin's well-recognized ability to induce the co-stimulation of glucose transport and fatty acid oxidation is notably incommensurate with the commonly presumed activation of the “Crabtree effect” (i.e., increased glycolysis induced by decreased mitochondrial) [19, 20] in response to metformin treatment [21-23]. Fatty acid oxidation cannot occur when mitochondrial function is interrupted in physiological conditions, such as anaerobiosis. Glycolysis, the only remaining energy pathway available, is therefore enhanced by the AMPK-directed stimulation of glucose transport. Because the presence of metformin and the activation of AMPK similarly correlate with the stimulation of glucose uptake, it has been erroneously assumed that this is caused by metformin's ability to reduce mitochondrial activity and increase glycolysis. However, metformin can operate aerobically because it stimulates glucose uptake and fatty acid oxidation [24]. Fryer et al. [25] have demonstrated in muscle cells that metformin can activate AMPK in the absence of any increase in the AMP-to-ATP ratio (AMP/ATP). Hawley et al. [12] have reported that metformin can increase AMPK activity in the absence of an increased AMP/ATP ratio in both Chinese hamster ovary fibroblasts and rat hepatoma cells. Zhang et al. [26] have confirmed that metformin can activate AMPK in the heart by increasing cytosolic AMP concentrations (metabolically active AMP) without altering the total AMP levels or the total AMP/ATP ratio. Moreover, the ablation of adenylate kinase expression (the phosphotransferase that catalyzes the conversion of two ADP molecules to AMP and ATP) in muscle cells does not affect metformin's activating effects on AMPK [24]. This result suggests that any mechanism involving increased AMP formation driven by ATP turnover (and hence, increased ADP formation) is improbable. In this complex scenario and as auspiciously suggested by Ouyang et al. [24], *“it is likely that the response of the cell to energy interruption is a distinct means of AMPK activation from the response to metformin”*. These authors have recently concluded that metformin's unique ability to activate AMPK while leading to the increased utilization of both major energy fuels, glucose and fat, occurs because metformin inhibits AMP deaminase (AMPD), the enzyme that converts AMP to ammonia and inosine monophosphate (IMP), the first fully formed nucleotide [24]. Therefore, at least in muscle cells, metformin may also activate AMPK via the prevention of AMPD-catalyzed AMP breakdown.

Metformin's insulin- and glucose-lowering effects are the non-cell autonomous systemic activities that are commonly claimed to be responsible for metformin's anticancer effects. Another of metformin's commonly disregarded non-cell autonomous systemic activities relates to the reported vitamin B12 decrease that may be observed following long-term treatment with metformin [27-32]. Up to 30% of patients taking metformin on a continuous basis have displayed reduced vitamin B12 absorption, and some rare cases of megaloblastic anemia have been reported in patients undergoing long-term metformin treatment. However, few studies have addressed the mechanism by which vitamin B12 metabolism is affected by metformin and the ultimate underlying mechanism behind metformin's antifolate activity, which has been a controversial subject. Metformin is known to elevate the levels of homocysteine, a surrogate marker of functional folate status. However, the evidence regarding the degree and method by which metformin depletes folate levels is inconclusive [33-36]. Diabetic patients are known to exhibit alterations in small bowel motility and bacterial overgrowth, and the latter has been hypothesized as the mechanism through which metformin induces B12 malabsorption [37]. Because the B12-intrinsic factor complex uptake by the ileal cell surface receptor is known to be calcium-dependent, the calcium-dependent membrane actions of metformin may affect the reduced vitamin B12 absorption in the absence of bacterial overgrowth [38]. Garcia & Tisma [39] have suggested that the enhanced pathological complete response of breast tumors in diabetic patients treated with both neoadjuvant chemotherapy and metformin occurs due to an unrecognized modulation of chemotherapy cytotoxicity by concurrent vitamin B12deficiency, which is initially imposed by chronic metformin exposure and is further potentiated by neoadjuvant chemotherapy-induced vitamin B12 decreases. However, the suggestion that alterations in folate metabolism/homeostasis cause metformin's anticancer activity lacks any mechanistic basis or experimental support.

We recently hypothesized that the use of liquid chromatography coupled with mass spectrometry to examine the effects of metformin on the tumor cells’ metabolome may help us to understand any occurrence of metformin's antifolate activity at the cancer cell-autonomous level. We predicted that the following would be true: 1.) because Vitamin B12 is a key cofactor of folate cycle enzymes, and folic acid has been shown to be active as a one-carbon unit donor in a number of metabolic processes, including histidine degradation and *de novo* nucleotide synthesis, any antifolate-like activity of metformin should translate into the detectable accumulation of formyl (CHO) or formimino (-CH-NH) intermediates, which operate as carriers of activated one-carbon units in a folate-dependent manner [40-45]; 2.) if metformin's anticancer activity is functionally associated with its ability to alter the *de novo* maintenance of folate-driven intracellular nucleotide pools, cancer cell growth should be efficiently restored after supplying the extracellular milieu with pre-formed nucleotide precursors [46-49]; 3.) if the recently described function of the DNA repair protein ataxia telangiectasia mutated (ATM) kinase and downstream activation of AMPK [50-56] is indeed secondary to metformin's ability to alter the carbon flow through the one carbon-related *de novo* maintenance of intracellular nucleotide pools, the ATM/AMPK tumor suppressive axis would be refractory to metformin's activating effects as long as the medium contains appropriate ready-formed thymidine and/or purines.

Here, we provide the first experimental evidence demonstrating that metformin can inhibit cancer cell growth by functionally mimicking the effects of a multi-targeted antifolate that secondarily induces the tumor-suppressor ATM/AMPK axis by altering the carbon flow through folate-dependent one-carbon metabolic pathways including the *de novo* maintenance of intracellular nucleotide pools.

## RESULTS

### Metabolite level analysis demonstrates that one-carbon metabolism and the associated glutathione pathway are significantly altered in metformin-treated breast cancer cells

**Figure 1:**
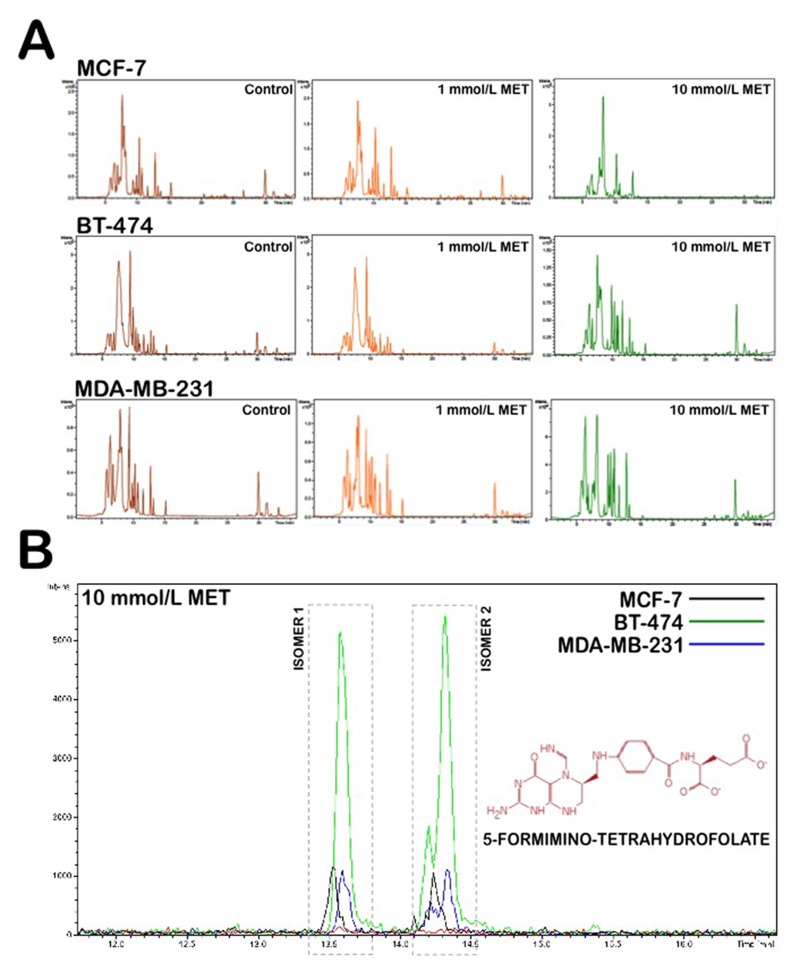
Application of nano-HPLC-ESI-QTOF-MS for the metabolomic analysis of metformin-treated breast cancer cells (**A**) (-)-nano-HPLC/ESI QTOF mass spectra derived from MCF-7, BT-474, and MDA-MB-231 breast cancer cell lines treated with 1 mmol/L and 10 mmol/L metformin. (**B**) Accumulation of 5-formimino-tetrahydrofolate following treatment with metformin in breast cancer cells. Figure shows an enlarged section of the spectra acquired on aqueous extracts of breast cancer cells following 48 h of treatment with either solvent control or 10 mmol/L metformin.

**Figure 2:**
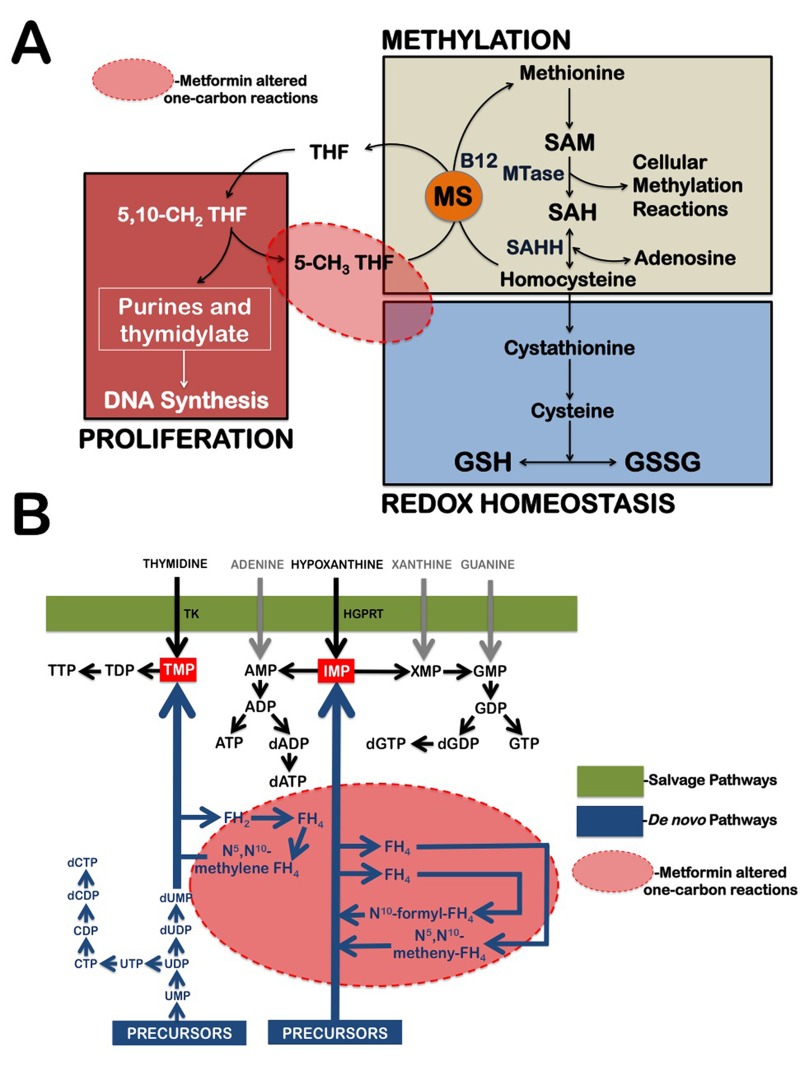
Metformin treatment alters carbon flow through folate-related one-carbon metabolic pathways (**A**) Diagram of tetrahydrofolate (THF)-dependent pathway of methionine trans-methylation to homocysteine and the trans-sulfuration pathway from homocysteine to glutathione (GSH) synthesis. MS, methionine synthase; SAH, S-adenosylhomocysteine; SAHH, SAH hydrolase; GSSG, oxidized glutathione disulfide; SAM, S-adenosylmethionine; MTase, methyltransferase. (**B**) Schematic depiction of purine and pyrimidine nucleotide biosynthesis through the de novo and salvage pathways. Because the biosynthetic pathways within the one-carbon network compete for a limiting pool of folate cofactors and folate-binding proteins can serve as “sinks” that sequester specific folates and thereby inhibit folate-dependent pathways, metformin-induced sequestration of 5-formimino-THF can simultaneously impairs the de novo nucleotide biosynthetic pathway while promoting GSH depletion. The conversion of thymidine and hypoxanthine to nucleotides via the folate-independent salvage pathway can partially rescue breast cancer cells from metformin's toxicity. TK, Thymidylate Kinase; HGPRT, hypoxanthine-guanine phosphoribosyltrans-ferase.

Three breast cancer cell lines, MCF-7, BT-474 and MDA-MB-231, were treated for 48 h with two different metformin doses (1 mmol/L and 10 mmol/L). To investigate the effect of metformin treatment on the breast cancer metabolome, we directly analyzed the patterns of “metabolite fingerprints” obtained from an ultra-performance liquid chromatography-electrospray ionization quadrupole time-of-flight mass spectrometry (UPLC-ESI-QTOF-MS) analysis [57-61]. The direct analysis of the nega- tive electrospray ionization fingerprints revealed that very few discriminatory metabolites were significantly and simultaneously altered in each of the three breast cancer cell lines in response to graded metformin concentrations (Fig. 1A). Specifically, the analysis of the spectra from the control and metformin treatments indicated that metformin treatment induced a notable accumulation of the one-carbon metabolism inter-mediate 5-formimino-tetrahydrofolate (5-formimino-THF) in each of the three breast cancer cell lines (Fig. 1B, Supplementary Fig. 1). In addition, all three breast cancer cell lines exhibited significant decreases in the reduced (GSH), oxidized disulfide (GSSG), and trisulfide (GSSSG) forms of glutathione in response to metformin treatment (Supplementary Fig. 2). Although they were apparently unrelated, these results may be consistent with an antifolate mode of action of metformin, which leads to disturbances in the inter-dependently linked one-carbon and glutathione metabolic processes (Fig. 2A) [62-64]. Tryptophan, another source of one-carbon units for purine biosynthesis *de novo*, also was concomitantly depleted in response to metformin (Supplementary Fig. 3). Similar conclusions were obtained when analyzing the ionization data acquired in the positive mode (data not shown).

### Reversal of the growth inhibition of metformin by thymidine and purines: Evidence for a multi-targeted, antifolate-like metformin activity

Cells produce ribonucleotides and deoxyribonucleotides *via* two pathways, *de novo* synthesis and salvage synthesis (Fig. 2B). Antifolates act by inhibiting the *de novo* biosynthesis of both thymidylate and purine nucleotides, whereas salvage synthesis refers to enzymatic reactions that convert free, pre-formed thymidine or purine bases into the corresponding nucleotides. Thymidine serves as a thymidylate (TMP) precursor in the pyrimidine salvage pathway when *de novo* synthesis is blocked by antifolates, such as aminopterine and methotrexate. Hypoxanthine is an inosine monophosphate (IMP) precursor in the purine salvage pathway when *de novo* synthesis is blocked by antifolates, such as aminopterine, methotrexate, and azaserine. If metformin's metabolomics-derived antifolate mode of action is accurate, it is likely that metformin will lose its growth-inhibitory activity against breast cancer cells while promoting the utilization of the pre-formed nucleotide precursors thymidine and hypoxanthine.

**Figure 3:**
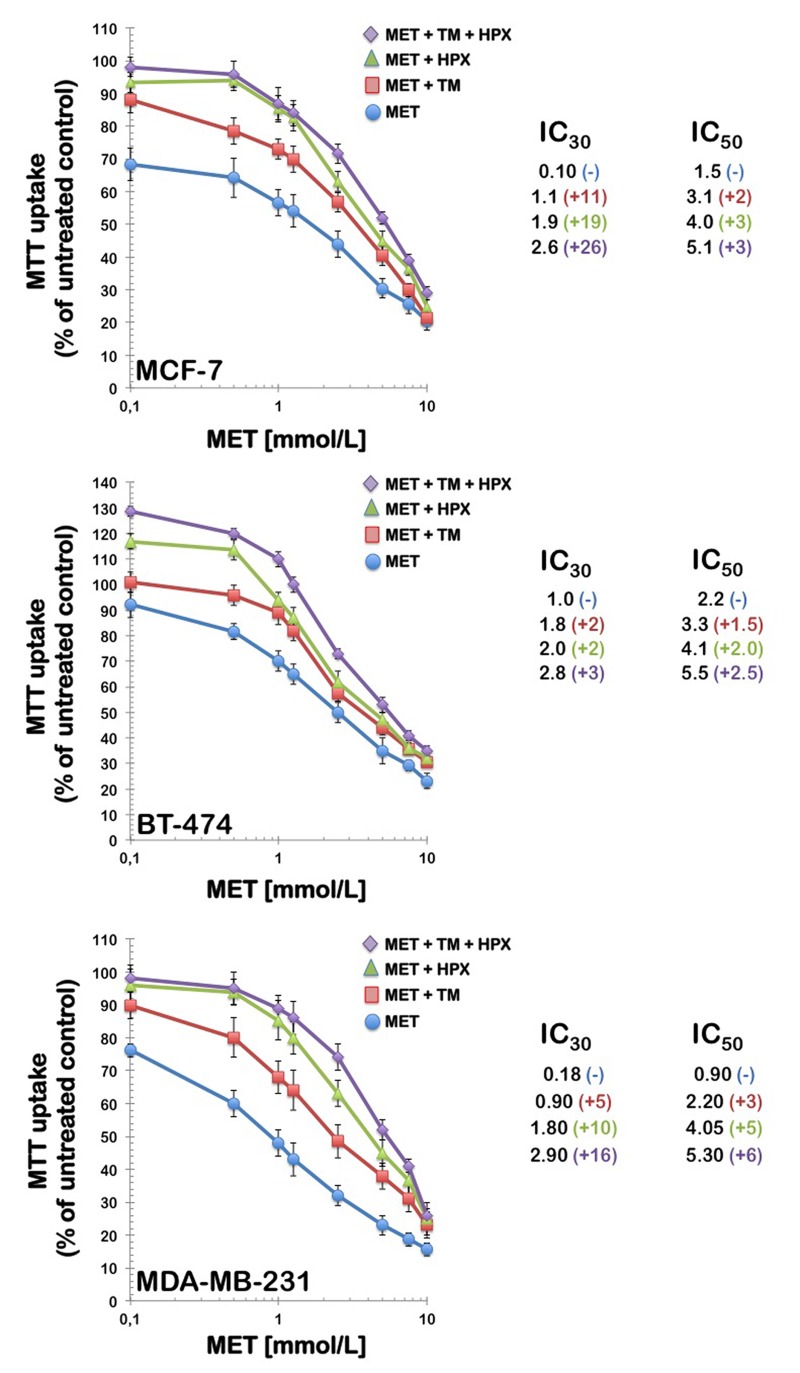
Partial reversal of the growth inhibition of metformin by thymidine and purines *Left*. MCF-7, BT-474, and MDA-MB-231 breast cancer cells were treated with the indicated concentrations of metformin alone or in the presence of thymidine (5.6 μmol/L), hypoxanthine (32 μmol/L), or a combination of thymidine with hypoxanthine. Metformin and modifying agents were added simultaneously and cell growth was determined after 96 h relative to controls without drug processed strictly in parallel using MTT-based cell viability assays. *Right*. The degree of resistance to metformin induced by thymidine and/or hypoxanthine was evaluated by dividing the IC30 and IC50 values obtained when cells were co-exposed to metformin and pre-formed nucleotides by those obtained in matched control cells cultured in the absence of an exogenous supply of thymidine and/or hypoxanthine. MET, Metformin; TM, Thymidine; HPX, Hypoxanthine.

To evaluate whether breast cancer cells can compensate for the antifolate-induced cancer cell toxicity effect of metformin by importing thymidine and purine bases from the extracellular environment, thymidine and/or hypoxanthine were exogenously added to the culture medium at the indicated concentrations in either the absence or presence of graded metformin concentrations. All of the cell cultures were incubated for 4 days, and the number of viable cells was determined using MTT-based assays (Fig. 3). The control experiments showed that the addition of 5.6 μmol/L thymidine and/or 32 μmol/L hypoxanthine did not affect the growth of breast carcinoma cells in the absence of metformin (data not shown). Of note, the inclusion of thymidine alone in the culture medium reduced the growth-inhibitory effects of metformin, thereby shifting the concentrations of the drug required to affect breast cancer cell growth by up to 11-fold the IC30 level and up to 3-fold at the IC_50_ level.

Additionally, the inclusion of hypoxanthine alone provided better protection than that obtained with thymidine, thereby shifting the concentrations of metformin required to affect breast cancer cell growth by up to 19-fold at the IC_30_ level and up to 5-fold at the IC_50_ level. Importantly, the inclusion of both thymidine and hypoxanthine reversed the effects of metformin even more significantly; the combination of thymidine plus hypoxanthine produced major rightward shifts in metformin's cytotoxic curves, which increased up to 26 and 6-fold the IC_30_ and IC_50_ metformin values, respectively.

### The AMPK kinase LKB1 does not mediate the salvage of pre-formed nucleotides when metformin inhibits cancer cell growth

Because loss-of-function mutations in the tumor suppressor gene product LKB1, which is the major upstream AMPK kinase, are commonly observed in human epithelial carcinomas [65-68], we sought to determine whether the antifolate-like growth-inhibitory effects of metformin remained intact in a LKB1-dependent or LKB1-independent manner. If LKB1 mediates the cell growth-inhibitory activation of AMPK in response to metformin's alteration of *de novo* nucleotide pool maintenance, we would expect that LKB1-negative cancer cannot compensate for the growth-inhibitory effect of metformin when importing thymidine and/or hypoxanthine from the extracellular milieu. We examined the dose-dependent anticancer activity of metformin in the absence and presence of exogenously supplemented thymidine plus hypoxanthine in LKB1-expressing human squamous carcinoma A431 cells [69] and HeLa cervical carcinoma cells bearing a biallelic deletion of LKB1 [70]. Supplementary Fig. 4 illustrates that metformin's growth-inhibitory effects were significantly altered by extracellular thymidine and hypoxanthine salvage regardless of the expression status of LKB1 in cancer cells.

### Metformin-induced activation of the tumor suppressive ATM/AMPK axis is secondary to metformin-induced alteration of *de novo* nucleotide pool maintenance

**Figure 4:**
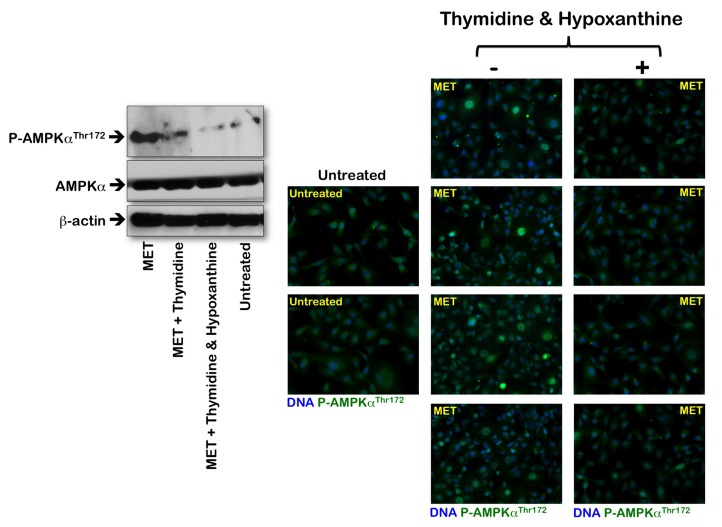
Effects of the folate-independent salvage pathway of nucleotide biosynthesis on metformin-induced activation of AMPK *Left*. Western blot analysis of total and phosphorylated AMPK (fosfo-AMPKα^Thr172^). Equal amounts of total protein (50 μg) were loaded in each line; use of β-actin as a control showed equal loading between lanes. Metformin (MET) was used at 1 mmol/L, thymidine was at 5.6 μmol/L, and hypoxanthine was at 32 μmol/L; drug exposure was 48 h. *Right*. Immunofluorescence for phospho-AMPKα^Thr172^ in MDA-MB-231 cells either untreated or treated with 1 mmol/L metformin (MET) in the absence or presence of thymidine plus hypoxanthine for 48 h. Images were captured in different channels for phospho-AMPKα^Thr172^ (green) and Hoechst 33258 (blue) with a 20x objective. (MET, metformin).

**Figure 5:**
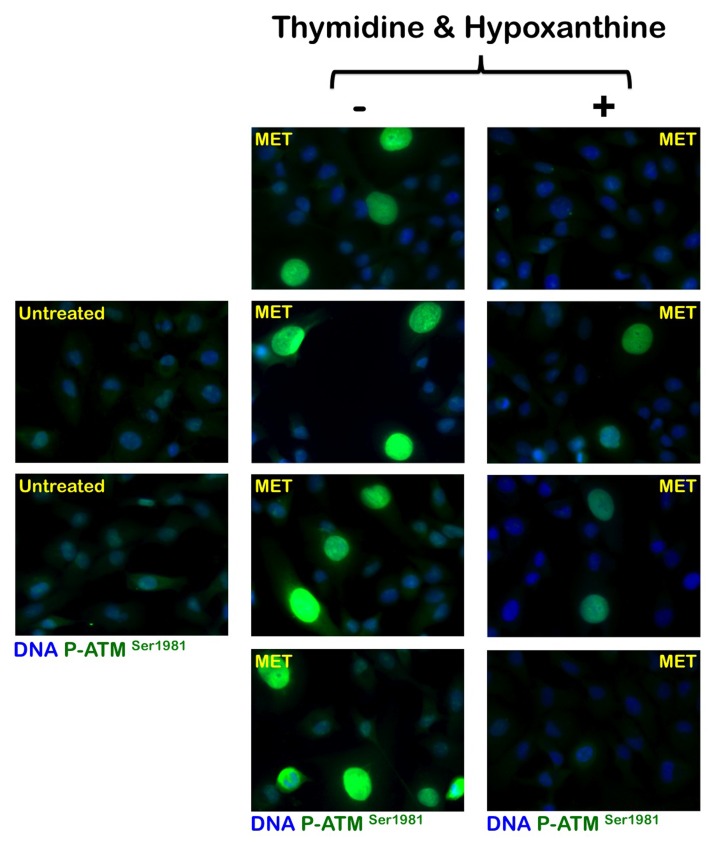
Effects of the folate-independent salvage path-way of nucleotide biosynthesis on metformin-induced activation of ATM Immunofluorescence for phospho-ATM^Ser1981^ in MDA-MB-231 cells either untreated or treated with 1 mmol/L metformin (MET) in the absence or presence of thymidine plus hypoxanthine for 48 h. Images were captured in different channels for phospho-ATMSer1981 (green) and Hoechst 33258 (blue) with a 20x objective.

To evaluate whether the activation status of the metabolic tumor suppressor AMPK in response to metformin was associated with altered *de novo* maintenance of intracellular nucleotide pools, we examined how an exogenous supply of thymidine and hypoxanthine impacted metformin's ability to activate AMPK (Fig. 4). Immunoblotting procedures confirmed that MDA-MB-231 cells likewise exhibited significant AMPKα phosphorylation levels upon metformin exposure. Of note, the activating phosphorylation of AMPKα returned to baseline levels similar to those detected in untreated control cells when the MDA-MB-231 cultures were treated with metformin in the presence of thymidine plus hypoxanthine. Metformin treatment significantly promoted the nuclear accumulation of fosfo-AMPKα^Thr172^ in MDA-MB-231 cells, whereas an exogenous supply of thymidine and hypoxanthine eliminated the nuclear accumulation of fosfo-AMPKα^Thr172^ in the presence of metformin (Fig. 4). We next evaluated whether the AMPK kinase ataxia telangiectasia mutated kinase (ATM) could also modify its metformin-regulated activation status in response to nucleotide supply (i.e., exogenous salvage). ATM autophosphorylation at residue Ser1981 was monitored by immunofluorescence microscopy 48 hours after metformin treatment in both the absence and presence of thymidine plus hypoxanthine (Fig. 5). The untreated controls displayed weak levels of fosfo-ATMSer1981 staining. Compared with the control samples, the metformin-treated (1 mmol/L) MDA-MB-231 cultures exhibited a spectrum of staining profiles varying from more punctate (visible fosfo-ATM^Ser1981^ foci) to a more homogeneous “pan-nuclear” staining profile that was not evident in the untreated controls. The yield of moderately and strongly nuclear staining of fosfo-ATM^Ser1981^ was found to be dependent on the disturbance of the *de novo* maintenance of intracellular nucleotide pools. Thus, the MDA-MB-231 cell cultures treated with metformin in the presence of an exogenous supply of thymidine and hypoxanthine notably failed to exhibit strong nuclear staining with fosfo-ATM^Ser1981^.

## DISCUSSION

Metformin has been repeatedly proposed to function as a *“mitochondrial poison”* by inhibiting complex I of the respiratory chain, which consequently leads to an imbalance of the AMP:ATP ratio, which is an extremely sensitive indicator of intracellular energy status that molecularly monitored by AMPK [13-16]. Because catabolism *“charges the battery”* by synthesizing ATP, while most other cellular processes consume ATP and tend to *“discharge the battery”*, AMPK's ability to detect and react to fluctuations in the AMP:ATP ratio ensures that the ATP synthesis rate closely aligns with the ATP consumption rate in most cells [13-16]. In the mitochondrial model describing metformin's activity, metformin-induced metabolic stress may cause the catabolic rate to be insufficient to respond to the ATP consumption rate, thereby causing ADP levels to rise and ATP levels to fall. ADP would be converted into AMP by adenylate kinase, and this process, when combined with the drop in ATP levels, would eventually active AMPK. Following AMPK activation, it is well known that catabolic pathways, such as fatty acid oxidation and glycolysis, become activated, whereas ATP-consuming pathways, such as lipogenesis, are inactivated. Although AMPK activation induces energy conservation and ultimately promotes the survival of normal cells, rapidly growing cancer cells cannot sustain this AMPK-induced limit on the utilization of available bioenergetic resources. Indeed, because the *“metabolic transformation”* of cancer cells commonly involves the oncogenetically driven addiction to nutrients (e.g., glucose) in the presence of genetically or functionally inactivated metabolic checkpoints, such as p53 deficiency, AMPK inactivation, and/or mTOR hyperactivation [71-73], it is not entirely unexpected that metformin-induced AMPK “re-activation” may induce specific tumor growth-inhibitory effects. However, we are now aware that the concept that metformin poisons mitochondrial respiratory enzyme activity can no longer be considered as the pivotal mechanism underlying metformin's ability to activate AMPK.

The unraveling of the mechanisms governing the alternative mitochondria-independent, AMPK-activating effects of metformin leading to cancer cell-autonomous growth inhibitory effects has just been initiated. In this regard, our current approach presents novel evidence that the disturbance of the one-carbon pool by folate is a previously unrecognized mechanism through which metformin can induce AMPK-associated breast cancer inhibitory actions at the cell-autonomous level. Metformin treatment was found to consistently increase the intracellular concentrations of the tetrahydrofolate (THF)-forming 5-formimino-THF, one of the last products of histidine metabolism that, upon the conversion to 5,10-methenyl-THF, links histidine to the folate cycle. Because histidine degradation, a metabolic pathway that is largely restricted to the liver and kidney tissues, has been extensively applied to the study of folate deficiency, it could be argued that the metformin-induced accumulation of 5-formimino-THF is caused by a previously unrecognized dependence of breast cancer cells upon folate-dependent histidine metabolism. Perhaps more importantly, the entry into the active one-carbon pool of intermediates involves 5,10-methylene-THF. This compound is used to synthesize thymidylate. Additionally, 5,10-methylene-THF is reduced to 5-methyl-THF during methionine biosynthesis, while it is oxidized to 10-formyl-THF in purine synthesis. The interconversion of 5,10-methylene-THF, 5,10-methenyl-THF, and 10-formyl-THF links the major source of single-carbon units, such as methylene-THF, with the synthesis of thymidylate (thymidylate synthase) or purines (5-phosphoribosylglycine-amide transformylase and 5-phosphoribosyl-5-aminoimidazole-4-carboxamide transformylase). Metformin notably mimics multi-targeted antifolate drugs such as pemetrexed, an antimetabolite that directly inhibits multiple folate-dependent target proteins and promotes the cytotoxic accumulation of metabolic intermediates of *de novo* purine biosynthesis that secondarily activates AMPK and inhibits mTOR [49, 74]. However, the salvage of extracellular thymidine and hypoxanthine facilitates a complete reversal from the cytotoxic effects of bona fide antifolates, such as pemetrexed. It is noteworthy that thymidine/hypoxanthine salvage rescue increases the concentrations of metformin required to elicit cytostatic (IC30 and IC50) effects, while thymidine/hypoxanthine salvage rescue is insufficient to modulate the cytotoxic action (i.e., the IC90 values) of metformin. Therefore, although cancer cells can acquire full refractoriness to higher concentrations of bona fide antifolates, such as methotrexate and pemetrexed, following exogenous supply with pre-formed nucleotides, the salvage of thymidine or preformed purines does not prevent metformin's cytotoxic activity at high concentrations (> 5 mmol/L). The modulation but not rescue of metformin toxicity by salvage metabolites strongly suggests that metformin's antifolate activity does not likely involve the direct inhibition of multiple enzyme targets in both purine and pyrimidine synthesis.

At low, clinically relevant concentrations, metformin apparently induces the cellular folate cofactors to become metabolically trapped as 5-formimino-THF. Metformin treatment causes folate to be “trapped” as 5-formimino-THF, which may significantly reduce the ability of the cancer cells to generate THF and other folate cofactors. Metformin treatment therefore promotes a cobalamin (Vitamin B12) deficiency-like effect at the cell-autonomous level that likely causes defects in *de novo* purine/pyrimidine biosynthesis and homocysteine accumulation; the latter is known to be associated with the blockade of glutathione (GSH) biosynthesis. GSH is a tripeptide of cysteine (Cys), glycine (Gly), and glutamate (Glu) that is synthesized *de novo* in all cells and serves as the major intracellular antioxidant and redox buffer. In this regard, the decreased levels of all GSH forms following metformin exposure likely results from the blockade of GSH biosynthesis at an early stage in the trans-sulfuration pathway upon homocysteine accumulation (Fig. 2) [75, 76]. Indeed, a severe depletion of GSH-like tripeptides such as Glu-Cys-Cys, Cys-Glu-Cys, Cys-Cys-Glu, and L-γ-glutamyl-L-alanyl-Gly (i.e. norophthalmic acid, an analogue of GSH with L-Cys replaced by L-Ala) and other disulfide-rich peptides (e.g. Arg-Arg-Cys-Lys) also took place in response to metformin treatment (data not shown). Nevertheless, the metabolic interdependency of the three interconnected pathways of folate, methionine, and GSH metabolism can explain that metformin treatment could translate into broader impact on DNA synthesis or repair and proliferation, cellular methylation, and GSH redox homeostasis.

The cell's inability to channel one-carbon units from this cofactor may result in the accumulation of one-carbon metabolism intermediates such as 5-formimino-THF at the expense of the forms used in DNA and RNA synthesis. Thus, cancer cells would exist in a *pseudo* folate-deficient or anemia-like state that significantly reduces the availability of folate intermediates in the pool of folate cofactors required for purine and pyrimidine synthesis, thereby ultimately decelerating or inhibiting DNA biosynthesis. Although we did not study the direct effects of metformin against purified folate cycle-related enzymes, our current findings do not contradict metformin's recently described ability to inhibit AMP deaminase in skeletal muscle cells [24] and to directly interact with AMPK by binding to the γ-regulatory subunit [77]. Because AMP deaminase activity (AMP → IMP + NH3) is the entry reaction to the purine nucleotide cycle, it is possible that the metformin-induced accumulation of 5-formimino-THF is secondary to the blockade of AMP deaminase. Although this possibility remains to be explored, the accumulation of intermediates in the folate-dependent metabolism of one-carbon units can exert potent inhibitory effects on the activities of AMP deaminase and adenosine deaminase [78-81]. The ability of metformin to impede the generation of the bioactive forms of folate and alter the *de novo* maintenance of intracellular nucleotide pools (i.e., a folate-associated mechanism responsive to exogenous thymidine and hypoxanthine) could reasonably occur at low metformin concentrations (higher metformin concentrations may exert folate-independent effects that are unresponsive to exogenous thymidine and hypoxanthine) by directly binding to γ-AMPK and making AMPK a more suitable substrate for other potential AMPK activators. Perhaps more importantly, our current description of metformin's antifolate activity and the blockade of AMP deaminase offers a novel molecular scenario that explains the mechanism by which metformin exerts anti-inflammatory activity. Metformin has been shown to reduce obesity-associated inflammation and other inflammatory responses, and it reduces serum C-reactive protein levels in women with polycystic ovary syndrome [82-86]. Additional mechanistic insight is required to conclude whether the alteration of the folate cycle directly relates to either metformin's ability to directly induce an anemia-like state or to directly or indirectly inhibit AMP deaminase. However, it is noteworthy that one consequence of AMP deaminase inhibition in response to perturbation of the folate cycle is increased AMP release into the extracellular space, where it can be converted by ecto-5'nucleotidase to adenosine. The latter is a labile but potent ligand of the family of receptors that regulates many different cellular and organ functions, including inflammation [87-90].

Metformin has been shown to exhibit antiproliferative and antineoplastic effects associated with the inhibition of mammalian target of rapamycin complex 1 (mTORC1) [91-94]. Larsson et al. [95] have recently revealed that metformin selectively inhibits the translation of mRNAs encoding cell-cycle proteins that promote neoplastic proliferation via the mTORC1/eukaryotic translation initiation factor 4E-binding protein pathway. How does metformin's DNA biosynthesis-associated antifolate activity promote the inhibition of mTOR-regulated protein translation? The perturbation of folate-mediated one-carbon metabolism by the multi-targeted antifolate pemetrexed has been demonstrated to efficiently activate AMPKα at Thr172; this process subsequently inhibits the mammalian target of rapamycin (mTOR) and the hypo-phosphorylation of the downstream targets of mTOR that control the initiation of protein synthesis and cell growth [49, 74]. Unlike AMP, metformin is not a direct allosteric AMPK activator, and it fails to activate AMPK in cell-free assays via the upstream kinases AMPKK1 and AMPKK2, which phosphorylate Thr172 in both the absence and presence of AMP [12]. Although it is unclear whether metformin, as other anticancer antifolates, represents a pro-drug that must be modified inside the intact cell into an active form, metformin may also act on upstream kinases other than AMPKK1 and AMPKK2. Metformin has been recently identified to activate the DNA repair protein ATM kinase, and this pivotal mechanism dictates the responsiveness of type 2 diabetic patients to the glucose-lowering effects of metformin. This finding has provided a novel molecular bridge causally linking ATM's metformin-regulated activation status with the ever-growing number of epidemiological and experimental studies suggesting that metformin confers anticancer activity [50-56]. Because the activation of the ATM-regulated DNA-damage response (DDR) network machinery is one of the earliest molecular events in the multistep progression of human epithelial carcinomas that impedes their evolution toward invasive malignancy, the selective activation of ATM with respect to DNA damage repair surveillance may directly contribute to metformin's cancer-preventive properties. Of note, our current findings demonstrate that low metformin concentrations cannot activate the ATM/AMPK tumor suppressive axis when cancer cells import thymidine and hypoxanthine from the extracellular milieu. Although mechanistic studies that unambiguously demonstrate a causal linkage between metformin's antifolate activity and direct AMPK activation by ATM are lacking, it is tempting to suggest that the metformin-altered *de novo* maintenance of intracellular nucleotide pools may lead to the phosphorylation of AMPK in an ATM-dependent and LKB1-independent manner [96-99]. Therefore, ATM may function as a potential AMPK kinase in response to the antifolate activity of metformin. Recently, 3-omic (i.e., transcriptomic, proteomic, and metabolomic) integration of ATM-mediated gene and protein expression and metabolite products has unexpectedly revealed that ATM dictates the purine, pyrimidine, and urea cycle pathways by regulating AMPK [100]. Of note, the data presented by Cheema et al. [100] have revealed that a major function of ATM induces imbalances in AMP and xanthine (i.e., metabolites associated with the folate-dependent purine metabolism pathway). Our findings provide new insight into ATM's ability to trigger AMPK-related metabolic responses beyond the ATM's function in DNA break repair. Caffeine, an AMPK activator, has been shown to sensitize cancer cells to the growth inhibitory effects of the antifolate pemetrexed by potentiating pemetrexed's ability to induce ATM phosphorylation [101]. Using similar approaches, our preliminary experiments indicate that metformin synergistically interacts with pemetrexed to inhibit breast cancer cell growth (data not shown).

Because cancer cell lines in standard cell-culture medium are exposed to relatively high folate concentrations compared with the folate levels in human plasma [102], the metformin concentrations required to inhibit cancer cell growth in media with low folate levels are likely much lower than those required in standard folate medium. Therefore, metformin's ability to alter the carbon flow through the folate-related *de novo* maintenance of intracellular nucleotide pools may represent a major mechanism of metformin's anticancer effect at physiological folate substrate concentrations and metformin blood serum levels. How does one then reconcile metformin's cancer-preventive benefits with a mechanism of action that actually mimics that of pemetrexed, a multi-targeted antifolate that is only effective due to its extreme toxicity? The relative contributions of the *de novo* and salvage pathways to nucleotide pool maintenance vary in different cells and tissues. Proliferating cells, including cancer cells, usually require a functional purine and pyrimidine *de novo* pathway to sustain their increased nucleotide demands. Indeed, this is the basis of the use of antifolate drugs in chemotherapy against cancer cells, which generally have higher DNA turnover [103-105]. It could be argued that the antifolate activity of metformin, although largely restricted to tumor cells requiring higher nucleotide concentrations, may also affect rapidly dividing cells, such as those within the bone marrow, and promote neural tube defects associated with folic acid deficiency (e.g., spina bifida and anencephaly). In this regard, few reports have described reversible megaloblastic anemia in individuals who have taken metformin for five years or more [29]. One study reported neural tube closure defects in rat fetuses exposed to metformin [106]. The “soft” character of metformin as an anticancer antifolate likely reflects a mechanism of action that, although involving a significant alteration of the carbon flow through folate-dependent one-carbon metabolism, does not imply direct inhibition of the folic acid cycle enzymes targeted by methotrexate, aminopterine or pemetrexed, thereby inducing significantly fewer side effects. Importantly, metformin's previously unrecognized antifolate growth inhibitory activity at the cancer cell-autonomous level reinforces its chemopreventive nature. We recently proposed that metformin, by activating an ATM-mediated DDR, may function as a “tissue sweeper” that significantly decreases the accumulation of dysfunctional, pre-malignant cells, including those with the ability to initiate and propagate tumors (i.e., cancer stem cells) [4, 52, 107, 108]. Because one ubiquitous event in cancer metabolism is the early, constitutive activation of *de novo* nucleotide biosynthesis and one-carbon metabolism may influence cancer mortality due to the critical role it plays in DNA synthesis and methylation, metformin's antifolate activity may silently operate to eliminate genetically damaged cells, initiated cells, or malignant cells while sparing their normal counterparts [109]. This mode of action is consistent with the understanding that although folate is generally anti-neoplastic before tumor foci are established, it may enhance tumor proliferation and progression after the tumor is established [110, 111]. Accordingly, population-based studies have suggested that one-carbon metabolism is an important pathway that may be targeted to improve the survival of cancer patients [112].

Corollary. Metronomic chemotherapy is defined as the continuous administration of chemotherapy at relatively low, minimally toxic doses at a frequent administration schedule at close regular intervals with no prolonged drug-free breaks. Although metformin's recently recognized anticancer effects are commonly referred to as *“new applications for an old drug”*, our current findings suggest that metformin may have long inadvertently functioned as an antifolate (the oldest of the antimetabolite class of anticancer agents) in millions of diabetic individuals. The observed reduction in cancer risk and mortality of diabetic patients chronically treated with the biguanide metformin may therefore represent an unintended metronomic chemotherapy approach targeting the differential utilization of *de novo* one-carbon metabolism by pre-malignant and malignant cells. Low-dose chemotherapy drugs (usually administered at one-tenth to one-third of the conventional maximum tolerated dose) that are administered continuously and frequently have been repeatedly shown to suppress vessel growth in tumor tissues and prevent the repair of damaged vascular endothelia [113-116]. It is unclear whether, by functioning as a bona fide *“low-dose metronomic chemotherapeutic”*, the antifolate metformin may lead to tumor suppression by devascularizing early tumor lesions. Metronomic metformin may also operate in a folate-related manner by promoting the senescence [117] or death of damaged cells or by preventing systemic genomic damage, increased DNA synthesis, cellular hyperproliferation and disruption of the DNA repair pathways associated with carcinogenesis-facilitating chronic inflammation [118-122] in patients with metabolic disorders.

## MATERIALS AND METHODS

### Chemicals

Acetic acid and acetonitrile for HPLC were obtained from Fluka, Sigma-Aldrich (Steinheim, Germany) and Lab-Scan (Gliwice, Sowinskiego, Poland), respectively. Methanol and chloroform used for metabolite extraction were also obtained from Lab-Scan. Water was purified using a Milli-Q system that was obtained from Millipore (Bedford, MA, USA).

### Sample preparation for the UPLC-ESI-QTOF-MS analysis

The frozen breast cancer cell pellets were subjected to extraction with the sequential use of aqueous (chilled water) and organic (chilled methanol and chloroform) solvents in a ratio of 1:4:3:2 (water:methanol:chloroform:water). The resulting aqueous extracts were treated with 500 L of chilled acetonitrile; they were vortex-mixed, stored at 4°C for 20 min to allow for protein precipitation, and centrifuged at 10000 g at 4°C for 10 min. The supernatants were evaporated in a vacuum concentrator and then dissolved in 100 μL of mobile phase A.

### UPLC-ESI-QTOF-MS analyses

The analyses were performed using a Waters Acquity UPLC system (Waters, Millford, MA). A Zorbax Eclipse Plus C18 (1.8 μm, 150 × 4.6 mm) UPLC column was used. The separation was performed at 25°C with a gradient elution program at a flow rate of 0.2 mL/min, and the injection volume was 5 μL. The mobile phases consisted of water with 0.5% of acetic acid (A) and acetonitrile (B). The following multi-step linear gradient was applied: 0 min, 5% B; 5 min, 15% B; 25 min, 30% B; 35 min, 95% B; and 40 min, 5% B. The initial conditions were held for 5 min.

The UPLC system was coupled to a micrOTOF-Q II mass spectrometer (Bruker Daltonik, Bremen, Germany) equipped with an ESI interface operating in negative and positive ion mode using a capillary voltage of +4 kV. The other optimum values of the ESI-QTOF parameters were as follows: drying gas temperature, 210°C; drying gas flow, 8 L/min, and nebulizing gas pressure, 2 bars. The detection was carried out considering a mass range of 50-1100 m/z. The collision energy values for the MS/MS experiments were adjusted as follows: m/z 100, 20 eV; m/z 500, 30 eV; and m/z 1000, 35 eV. Nitrogen was used as a drying, nebulizing and collision gas.

During the development of the method, external instrument calibration of the mass spectrometer was performed using a 74900-00-05 Cole Palmer syringe pump (Vernon Hills, Illinois, USA) directly connected to the interface and a sodium acetate cluster solution (5 mmol/L sodium hydroxide and 0.2% acetic acid in water:isopropanol (1:1, v/v)). The calibration solution was injected at the beginning of each run, and each spectrum was calibrated prior to compound identification. By using this method, an exact calibration curve based on numerous cluster masses that each differed by 82 Da (NaC_2_H_3_O_2_) was obtained. Due to the compensation of temperature drift in the micrOTOF-Q II, this external calibration provided accurate mass values (better than 5 ppm) for a complete run without the need for a dual sprayer setup for internal mass calibration.

The accurate mass data of the molecular ions were processed through the software program Data Analysis 4.0 (Bruker Daltonik, Bremen, Germany), which provided a list of possible elemental formulas using the Smart Formula algorithm. If no other elements are specified, the Smart Formula algorithm assumes a CHNO distribution, and it provides standard functionalities, such as minimum/maximum elemental range, electron configuration, ring-plus double-bond equivalents, and a sophisticated comparison of the theoretical and measured isotope patterns (sigma value) to promote increased confidence in the suggested molecular formula.

### Identification of metabolites

Metabolites were identified by interpretation of their MS and MS/MS spectra combined with the use of metabolite databases. The high mass accuracy and true isotopic pattern in both MS and MS/MS spectra provided by QTOF-MS analyser enabled to establish the most probable molecular formulas for every metabolite and their corresponding fragments. These molecular formulas were matched against available databases, namely Metlin, Human Metabolome Database, and Scifinder. Finally the possible structures provided by the databases were corroborated or rejected in the basis of their fragmentation patterns.

### Cell lines and culture conditions

MCF-7, BT-474, and MDA-MB-231 breast cancer cell lines were obtained from the American Type Culture Collection (ATCC) and they were routinely grown in Dulbecco's modified Eagle's medium (DMEM, Gibco) containing 10% heat-inactivated fetal bovine serum (FBS, Bio-Whittaker), 1% L-glutamine, 1% sodium pyruvate, 50 U/mL penicillin and 50 μg/mL streptomycin. Cells were maintained at 37°C in a humidified atmosphere of 95% air/5% CO2. Cells were screened periodically for Mycoplasma contamination.

### Metabolic status assessment (MTT-based cell viability assays)

The ability of the folate-independent salvage pathway of nucleotide biosynthesis to modulate metformin reduced breast cancer cell viability was monitored by using a standard colorimetric MTT (3-4, 5-dimethylthiazol-2-yl-2, 5-diphenyl-tetrazolium bromide) reduction assay. Cells in exponential growth were harvested by trypsinization and seeded at a concentration of ~2.5 × 10_3_ cells/200 μL/well into 96-well plates, and allowed an overnight period for attachment. Then the medium was removed and fresh medium along with various concentrations of metformin, thymidine, hypoxanthine or combinations of the compounds were added to cultures in parallel, as specified. Agents were studied in combination concurrently. Control cells without agents were cultured using the same conditions with comparable media changes. Compounds were not renewed during the entire period of cell exposure. Following treatment (4 days), the medium was removed and replaced by fresh drug-free medium (100 μL/well), and MTT (5 mg/mL in PBS) was added to each well at a 1/10 volume. After incubation for 2-3 h at 37°C, the supernatants were carefully aspirated, 100 μL of DMSO were added to each well, and the plates agitated to dissolve the crystal product. Absorbances were measured at 570 nm using a multi-well plate reader. The cell viability effects from exposure of cells to each agent alone and their combination were analyzed as percentages of the control cell absorbances, which were obtained from control wells treated with appropriate concentrations of the agents’ vehicles that were processed simultaneously. For each treatment, cell viability was evaluated as a percentage using the following equation: *(A_570_ of treated sample/A_570_ of untreated sample) × 100*.

Breast cancer cell sensitivity to metformin was expressed in terms of the concentration of drug required to decrease by 30% and 50% cell viability (IC30 and IC50, respectively). Since the percentage of control absorbance was considered to be the surviving fraction of cells, the IC_30_ and IC_50_ values were defined as the concentration of metformin that produced 30% and 50% reduction in control absorbance (by interpolation), respectively. The degree of resistance to metformin by thymidine and/or hypoxanthine was evaluated by dividing IC_30_ and IC_50_ values of control cells by those obtained when cells were simultaneously exposed to an exogenous supply of pre-formed nucleotides.

### Immunoblotting

The activation of phosphorylation and total expression of AMPK was assessed using immunoblotting procedures and the AMPK and ACC (#9957) Antibody Kit (Cell Signaling Technology, Inc.). Briefly, metformin-treated and untreated control cells were washed twice with cold PBS and then lysed as described above. Equal amounts of protein (i.e., 50 μg) were resuspended in 5x Laemmli sample buffer (10 min at 70°C), subjected to 10% SDS-PAGE and transferred onto nitrocellulose membranes. The nitrocellulose membranes were blocked for 1 h at RT with TBS-T buffer [25 mmol/L TRIS- HCl (pH 7.5), 150 mmol/L NaCl, 0.05% Tween 20] containing 5% (w/v) nonfat dry milk to minimize non-specific binding. Subsequently, the treated membranes were washed in TBS-T and incubated with phospho-AMPKα^Thr172^ (Clone 40H9) or total AMPK antibodies, as specified, in 1x TBS-T buffer containing 5% w/v BSA and 0.1% Tween-20 at 4°C with gentle shaking overnight. The membranes were washed with TBS-T, incubated with horseradish peroxidase-conjugated secondary anti-rabbit IgG in TBS-T for 1 h, and the immunoreactive bands were detected using a chemiluminescence reagent (Pierce). The blots were re-probed with an antibody against β-actin to control for protein loading and transfer. Densitometric values of the proteins bands were quantified using Scion Image software (Scion Corporation).

### Immunofluorescence staining and high-content confocal imaging

Cells were seeded at approximately 5,000 cells/well in 96-well clear bottom imaging tissue culture plates (Becton Dickinson Biosciences; San Jose, CA) optimized for automated imaging applications. Triton® X-100 permeabilization and blocking, primary antibody staining, secondary antibody staining using Alexa Fluor® 488/594 goat anti-rabbit/mouse IgGs (Invitrogen, Probes, Eugene, Oregon) and counterstaining (using Hoechst 33258; Invitrogen) were performed following BD Biosciences protocols. Images were captured in different channels for Alexa Fluor® 488 (pseudocolored green) and Hoechst 33258 (pseudocolored blue) on a BD Pathway™ 855 Bioimager System (Becton Dickinson Biosciences, San Jose, California) with 20x or 40x objectives (NA 075 Olympus). Merging images were obtained according to the Recommended Assay Procedure using BD Attovision™ software.

## SUPPLEMENTARY FIGURES



## References

[R1] Martin-Castillo B, Vazquez-Martin A, Oliveras-Ferraros C, Menendez JA (2010). Metformin and cancer: doses, mechanisms and the dandelion and hormetic phenomena. Cell Cycle.

[R2] Vazquez-Martin A, Oliveras-Ferraros C, Cufí S, Martin-Castillo B, Menendez JA (2010). Metformin and energy metabolism in breast cancer: from insulin physiology to tumour-initiating stem cells. Curr Mol Med.

[R3] Jalving M, Gietema JA, Lefrandt JD, de Jong S, Reyners AK, Gans RO, de Vries EG (2010). Metformin: taking away the candy for cancer?. Eur J Cancer.

[R4] Del Barco S, Vazquez-Martin A, Cufí S, Oliveras-Ferraros C, Bosch-Barrera J, Joven J, Martin-Castillo B, Menendez JA (2011). Metformin: multi-faceted protection against cancer. Oncotarget.

[R5] Bost F, Sahra IB, Le Marchand-Brustel Y, Tanti JF (2012). Metformin and cancer therapy. Curr Opin Oncol.

[R6] Zakikhani M, Dowling R, Fantus IG, Sonenberg N, Pollak M (2006). Metformin is an AMP kinase-dependent growth inhibitor for breast cancer cells. Cancer Res.

[R7] Buzzai M, Jones RG, Amaravadi RK, Lum JJ, DeBerardinis RJ, Zhao F, Viollet B, Thompson CB (2007). Systemic treatment with the antidiabetic drug metformin selectively impairs p53-deficient tumor cell growth. Cancer Res.

[R8] Zakikhani M, Dowling RJ, Sonenberg N, Pollak MN (2008). The effects of adiponectin and metformin on prostate and colon neoplasia involve activation of AMP-activated protein kinase. Cancer Prev Res (Phila).

[R9] Hosono K, Endo H, Takahashi H, Sugiyama M, Uchiyama T, Suzuki K, Nozaki Y, Yoneda K, Fujita K, Yoneda M, Inamori M, Tomatsu A, Chihara T, Shimpo K, Nakagama H, Nakajima A (2010). Metformin suppresses azoxymethane-induced colorectal aberrant crypt foci by activating AMP-activated protein kinase. Mol Carcinog.

[R10] Rocha GZ, Dias MM, Ropelle ER, Osório-Costa F, Rossato FA, Vercesi AE, Saad MJ, Carvalheira JB (2011). Metformin amplifies chemotherapy-induced AMPK activation and antitumoral growth. Clin Cancer Res.

[R11] Hawley SA, Ross FA, Chevtzoff C, Green KA, Evans A, Fogarty S, Towler MC, Brown LJ, Ogunbayo OA, Evans AM, Hardie DG (2010). Use of cells expressing gamma subunit variants to identify diverse mechanisms of AMPK activation. Cell Metab.

[R12] Hawley SA, Gadalla AE, Olsen GS, Hardie DG (2002). The antidiabetic drug metformin activates the AMP-activated protein kinase cascade via an adenine nucleotide-independent mechanism. Diabetes.

[R13] Hardie DG (2011). Sensing of energy and nutrients by AMP-activated protein kinase. Am J Clin Nutr.

[R14] Hardie DG (2011). Adenosine Monophosphate-Activated Protein Kinase: A Central Regulator of Metabolism with Roles in Diabetes, Cancer, and Viral Infection. Cold Spring Harb Symp Quant Biol.

[R15] Hardie DG (2011). AMP-activated protein kinase: an energy sensor that regulates all aspects of cell function. Genes Dev.

[R16] Hardie DG (2006). Neither LKB1 nor AMPK are the direct targets of metformin. Gastroenterology.

[R17] Hoek JB (2006). Metformin and the fate of fat. Gastroenterology.

[R18] Liu J, Shen W, Zhao B, Wang Y, Wertz K, Weber P, Zhang P (2009). Targeting mitochondrial biogenesis for preventing and treating insulin resistance in diabetes and obesity: Hope from natural mitochondrial nutrients. Adv Drug Deliv Rev.

[R19] Ibsen KH (1961). The Crabtree effect: a review. Cancer Res.

[R20] Sussman I, Erecińska M, Wilson DF (1980). Regulation of cellular energy metabolism: the Crabtree effect. Biochim Biophys Acta.

[R21] Misra P (2008). AMP activated protein kinase: a next generation target for total metabolic control. Expert Opin Ther Targets.

[R22] Musi N (2006). AMP-activated protein kinase and type 2 diabetes. Curr Med Chem.

[R23] Collier CA, Bruce CR, Smith AC, Lopaschuk G, Dyck DJ (2006). Metformin counters the insulin-induced suppression of fatty acid oxidation and stimulation of triacylglycerol storage in rodent skeletal muscle. Am J Physiol Endocrinol Metab.

[R24] Ouyang J, Parakhia RA, Ochs RS (2011). Metformin activates AMP kinase through inhibition of AMP deaminase. J Biol Chem.

[R25] Fryer LG, Parbu-Patel A, Carling D (2002). The Anti-diabetic drugs rosiglitazone and metformin stimulate AMP-activated protein kinase through distinct signaling pathways. J Biol Chem.

[R26] Zhang L, He H, Balschi JA (2007). Metformin and phenformin activate AMP-activated protein kinase in the heart by increasing cytosolic AMP concentration. Am J Physiol Heart Circ Physiol.

[R27] Stowers JM, Smith OA (1971). Vitamin B 12 and metformin. Br Med J.

[R28] Herbert V (1972). Metformin and B-12 malabsorption. Ann Intern Med.

[R29] Callaghan TS, Hadden DR, Tomkin GH (1980). Megaloblastic anaemia due to vitamin B12 malabsorption associated with long-term metformin treatment. Br Med J.

[R30] Adams JF, Clark JS, Ireland JT, Kesson CM, Watson WS (1983). Malabsorption of vitamin B12 and intrinsic factor secretion during biguanide therapy. Diabetologia.

[R31] Liu KW, Dai LK, Jean W (2006). Metformin-related vitamin B12 deficiency. Age Ageing.

[R32] Andrès E, Federici L (2007). Vitamin B12 deficiency in patients receiving metformin: clinical data. Arch Intern Med.

[R33] Carlsen SM, Følling I, Grill V, Bjerve KS, Schneede J, Refsum H (1997). Metformin increases total serum homocysteine levels in non-diabetic male patients with coronary heart disease. Scand J Clin Lab Invest.

[R34] Aarsand AK, Carlsen SM (1998). Folate administration reduces circulating homocysteine levels in NIDDM patients on long-term metformin treatment. J Intern Med.

[R35] Sahin M, Tutuncu NB, Ertugrul D, Tanaci N, Guvener ND (2007). Effects of metformin or rosiglitazone on serum concentrations of homocysteine, folate, and vitamin B12 in patients with type 2 diabetes mellitus. J Diabetes Complications.

[R36] Palomba S, Falbo A, Giallauria F, Russo T, Tolino A, Zullo F, Colao A, Orio F (2010). Effects of metformin with or without supplementation with folate on homocysteine levels and vascular endothelium of women with polycystic ovary syndrome. Diabetes Care.

[R37] Caspary WF, Zavada I, Reimold W, Deuticke U, Emrich D, Willms B (1977). Alteration of bile acid metabolism and vitamin-B12-absorption in diabetics on biguanides. Diabetologia.

[R38] Bauman WA, Shaw S, Jayatilleke E, Spungen AM, Herbert V (2000). Increased intake of calcium reverses vitamin B12 malabsorption induced by metformin. Diabetes Care.

[R39] Garcia A, Tisman G (2010). Metformin, B(12), and enhanced breast cancer response to chemotherapy. J Clin Oncol.

[R40] Kamen B (1997). Folate and antifolate pharmacology. Semin Oncol.

[R41] Lucock M (2000). Folic acid: nutritional biochemistry, molecular biology, and role in disease processes. Mol Genet Metab.

[R42] Stover PJ (2004). Physiology of folate and vitamin B12 in health and disease. Nutr Rev.

[R43] Ulrich CM, Reed MC, Nijhout HF (2008). Modeling folate, one-carbon metabolism, and DNA methylation. Nutr Rev.

[R44] Xu X, Chen J (2009). One-carbon metabolism and breast cancer: an epidemiological perspective. J Genet Genomics.

[R45] Stover PJ, Field MS (2011). Trafficking of intracellular folates. Adv Nutr.

[R46] Refsum H, Christensen B, Djurhuus R, Ueland PM (1991). Interaction between methotrexate, “rescue” agents and cell proliferation as modulators of homocysteine export from cells in culture. J Pharmacol Exp Ther.

[R47] Smith PG, Marshman E, Calvert AH, Newell DR, Curtin NJ (1999). Prevention of thymidine and hypoxanthine rescue from MTA (LY231514) growth inhibition by dipyridamole in human lung cancer cell lines. Semin Oncol.

[R48] Thomas HD, Saravanan K, Wang LZ, Lin MJ, Northen JS, Barlow H, Barton M, Newell DR, Griffin RJ, Golding BT, Curtin NJ (2009). Preclinical evaluation of a novel pyrimidopyrimidine for the prevention of nucleoside and nucleobase reversal of antifolate cytotoxicity. Mol Cancer Ther.

[R49] Racanelli AC, Rothbart SB, Heyer CL, Moran RG (2009). Therapeutics by cytotoxic metabolite accumulation: pemetrexed causes ZMP accumulation, AMPK activation, and mammalian target of rapamycin inhibition. Cancer Res.

[R50] Zhou K, Bellenguez C, Spencer CC, Bennett AJ, Coleman RL, Tavendale R, Hawley SA, Donnelly LA, Schofield C, Groves CJ, Burch L, Carr F, Strange A, Freeman C, Blackwell JM, Bramon E, Brown MA, Casas JP, Corvin A, Craddock N, Deloukas P, Dronov S, Duncanson A, Edkins S, Gray E, Hunt S, Jankowski J, Langford C, Markus HS, Mathew CG, Plomin R, Rautanen A, Sawcer SJ, Samani NJ, Trembath R, Viswanathan AC, Wood NW, Harries LW, Hattersley AT, Doney AS, Colhoun H, Morris AD, Sutherland C, Hardie DG, Peltonen L, McCarthy MI, Holman RR, Palmer CN, Donnelly P, Pearson ER, GoDARTS and UKPDS Diabetes Pharmacogenetics Study Group; Wellcome Trust Case Control Consortium 2, MAGIC investigators (2011). Common variants near ATM are associated with glycemic response to metformin in type 2 diabetes. Nat Genet.

[R51] Vazquez-Martin A, Oliveras-Ferraros C, Cufí S, Martin-Castillo B, Menendez JA (2011). Metformin activates an ataxia telangiectasia mutated (ATM)/Chk2-regulated DNA damage-like response. Cell Cycle.

[R52] Menendez JA, Cufí S, Oliveras-Ferraros C, Martin-Castillo B, Joven J, Vellon L, Vazquez-Martin A (2011). Metformin and the ATM DNA damage response (DDR): accelerating the onset of stress-induced senescence to boost protection against cancer. Aging (Albany NY).

[R53] van Leeuwen N, Nijpels G, Becker ML, Deshmukh H, Zhou K, Stricker BH, Uitterlinden AG, Hofman A, van 't Riet E, Palmer CN, Guigas B, Slagboom PE, Durrington P, Calle RA, Neil A, Hitman G, Livingstone SJ, Colhoun H, Holman RR, McCarthy MI, Dekker JM, 't Hart LM, Pearson ER (2012). A gene variant near ATM is significantly associated with metformin treatment response in type 2 diabetes: a replication and meta-analysis of five cohorts. Diabetologia.

[R54] Yee SW, Chen L, Giacomini KM (2012). The role of ATM in response to metformin treatment and activation of AMPK. Nat Genet.

[R55] Woods A, Leiper JM, Carling D (2012). The role of ATM in response to metformin treatment and activation of AMPK. Nat Genet.

[R56] Zhou K, Bellenguez C, Sutherland C, Hardie G, Palmer C, Donnelly P, Pearson E (2012). The role of ATM in response to metformin treatment and activation of AMPK. Nat Genet.

[R57] Sana TR, Waddell K, Fischer SM (2008). A sample extraction and chromatographic strategy for increasing LC/MS detection coverage of the erythrocyte metabolome. J Chromatogr B Analyt Technol Biomed Life Sci.

[R58] Sana TR, Roark JC, Li X, Waddell K, Fischer SM (2008). Molecular formula and METLIN Personal Metabolite Database matching applied to the identification of compounds generated by LC/TOF-MS. J Biomol Tech.

[R59] Liu A, Chen Y, Yang Z, Feng Y, Rui W, Luo W, Liu Y, Gonzalez FJ, Dai R (2009). New metabolites of fenofibrate in Sprague-Dawley rats by UPLC-ESI-QTOF-MS-based metabolomics coupled with LC-MS/MS. Xenobiotica.

[R60] Manna SK, Patterson AD, Yang Q, Krausz KW, Idle JR, Fornace AJ, Gonzalez FJ (2011). UPLC-MS-based urine metabolomics reveals indole-3-lactic acid and phenyllactic acid as conserved biomarkers for alcohol-induced liver disease in the Ppara-null mouse model. J Proteome Res.

[R61] Zhang X, Choi FF, Zhou Y, Leung FP, Tan S, Lin S, Xu H, Jia W, Sung JJ, Cai Z, Bian Z (2012). Metabolite profiling of plasma and urine from rats with TNBS-induced acute colitis using UPLC-ESI-QTOF-MS-based metabonomics - a pilot study. FEBS J.

[R62] Depeint F, Bruce WR, Shangari N, Mehta R, O'Brien PJ (2006). Mitochondrial function and toxicity: role of B vitamins on the one-carbon transfer pathways. Chem Biol Interact.

[R63] Reed MC, Thomas RL, Pavisic J, James SJ, Ulrich CM, Nijhout HF (2008). A mathematical model of glutathione metabolism. Theor Biol Med Model.

[R64] Nijhout HF, Reed MC, Ulrich CM (2008). Mathematical models of folate-mediated one-carbon metabolism. Vitam Horm.

[R65] Sanchez-Cespedes M (2007). A role for LKB1 gene in human cancer beyond the Peutz-Jeghers syndrome. Oncogene.

[R66] Jansen M, Ten Klooster JP, Offerhaus GJ, Clevers H (2009). LKB1 and AMPK family signaling: the intimate link between cell polarity and energy metabolism. Physiol Rev.

[R67] Herrmann JL, Byekova Y, Elmets CA, Athar M (2011). Liver kinase B1 (LKB1) in the pathogenesis of epithelial cancers. Cancer Lett.

[R68] van Veelen W, Korsse SE, van de Laar L, Peppelenbosch MP (2011). The long and winding road to rational treatment of cancer associated with LKB1/AMPK/TSC/mTORC1 signaling. Oncogene.

[R69] Byekova YA, Herrmann JL, Xu J, Elmets CA, Athar M (2011). Liver kinase B1 (LKB1) in the pathogenesis of UVB-induced murine basal cell carcinoma. Arch Biochem Biophys.

[R70] Wingo SN, Gallardo TD, Akbay EA, Liang MC, Contreras CM, Boren T, Shimamura T, Miller DS, Sharpless NE, Bardeesy N, Kwiatkowski DJ, Schorge JO, Wong KK, Castrillon DH (2009). Somatic LKB1 mutations promote cervical cancer progression. PLoS One.

[R71] Muñoz-Pinedo C, El Mjiyad N, Ricci JE (2012). Cancer metabolism: current perspectives and future directions. Cell Death Dis.

[R72] Deberardinis RJ, Sayed N, Ditsworth D, Thompson CB (2008). Brick by brick: metabolism and tumor cell growth. Curr Opin Genet Dev.

[R73] DeBerardinis RJ, Lum JJ, Hatzivassiliou G, Thompson CB (2008). The biology of cancer: metabolic reprogramming fuels cell growth and proliferation. Cell Metab.

[R74] Rothbart SB, Racanelli AC, Moran RG (2010). Pemetrexed indirectly activates the metabolic kinase AMPK in human carcinomas. Cancer Res.

[R75] Mosharov E, Cranford MR, Banerjee R (2000). The quantitatively important relationship between homocysteine metabolism and glutathione synthesis by the transsulfuration pathway and its regulation by redox changes. Biochemistry.

[R76] Bayet-Robert M, Morvan D, Chollet P, Barthomeuf C (2010). Pharmacometabolomics of docetaxel-treated human MCF7 breast cancer cells provides evidence of varying cellular responses at high and low doses. Breast Cancer Res Treat.

[R77] Zhang Y, Wang Y, Bao C, Xu Y, Shen H, Chen J, Yan J, Chen Y (2012). Metformin interacts with AMPK through binding to γ subunit. Mol Cell Biochem.

[R78] Baggott JE, Vaughn WH, Hudson BB (1986). Inhibition of 5-aminoimidazole-4-carboxamide ribotide transformylase, adenosine deaminase and 5’-adenylate deaminase by polyglutamates of methotrexate and oxidized folates and by 5-aminoimidazole-4-carboxamide riboside and ribotide. Biochem J.

[R79] Cutolo M, Sulli A, Pizzorni C, Seriolo B, Straub RH (2001). Anti-inflammatory mechanisms of methotrexate in rheumatoid arthritis. Ann Rheum Dis.

[R80] Chan ES, Cronstein BN (2010). Methotrexate--how does it really work?. Nat Rev Rheumatol.

[R81] Cronstein B (2010). How does methotrexate suppress inflammation?. Clin Exp Rheumatol.

[R82] Morin-Papunen L, Rautio K, Ruokonen A, Hedberg P, Puukka M, Tapanainen JS (2003). Metformin reduces serum C-reactive protein levels in women with polycystic ovary syndrome. J Clin Endocrinol Metab.

[R83] Bergheim I, Luyendyk JP, Steele C, Russell GK, Guo L, Roth RA, Arteel GE (2006). Metformin prevents endotoxin-induced liver injury after partial hepatectomy. J Pharmacol Exp Ther.

[R84] Luque-Ramírez M, Escobar-Morreale HF (2010). Treatment of polycystic ovary syndrome (PCOS) with metformin ameliorates insulin resistance in parallel with the decrease of serum interleukin-6 concentrations. Horm Metab Res.

[R85] Luque-Ramírez M, Escobar-Morreale HF (2010). Treatment of polycystic ovary syndrome (PCOS) with metformin ameliorates insulin resistance in parallel with the decrease of serum interleukin-6 concentrations. Horm Metab Res.

[R86] Fidan E, Onder Ersoz H, Yilmaz M, Yilmaz H, Kocak M, Karahan C, Erem C (2011). The effects of rosiglitazone and metformin on inflammation and endothelial dysfunction in patients with type 2 diabetes mellitus. Acta Diabetol.

[R87] Eltzschig HK, Weissmüller T, Mager A, Eckle T (2006). Nucleotide metabolism and cell-cell interactions. Methods Mol Biol.

[R88] Montesinos MC, Takedachi M, Thompson LF, Wilder TF, Fernández P, Cronstein BN (2007). The antiinflammatory mechanism of methotrexate depends on extracellular conversion of adenine nucleotides to adenosine by ecto-5’-nucleotidase: findings in a study of ecto-5’-nucleotidase gene-deficient mice. Arthritis Rheum.

[R89] Vass G, Horváth I (2008). Adenosine and adenosine receptors in the pathomechanism and treatment of respiratory diseases. Curr Med Chem.

[R90] Csóka B, Selmeczy Z, Koscsó B, Németh ZH, Pacher P, Murray PJ, Kepka-Lenhart D, Morris SM, Gause WC, Leibovich SJ, Haskó G (2012). Adenosine promotes alternative macrophage activation via A2A and A2B receptors. FASEB J.

[R91] Dowling RJ, Zakikhani M, Fantus IG, Pollak M, Sonenberg N (2007). Metformin inhibits mammalian target of rapamycin-dependent translation initiation in breast cancer cells. Cancer Res.

[R92] Vazquez-Martin A, Oliveras-Ferraros C, Menendez JA (2009). The antidiabetic drug metformin suppresses HER2 (erbB-2) oncoprotein overexpression via inhibition of the mTOR effector p70S6K1 in human breast carcinoma cells. Cell Cycle.

[R93] Vázquez-Martín A, Oliveras-Ferraros C, del Barco S, Martín-Castillo B, Menéndez JA (2009). mTOR inhibitors and the anti-diabetic biguanide metformin: new insights into the molecular management of breast cancer resistance to the HER2 tyrosine kinase inhibitor lapatinib (Tykerb). Clin Transl Oncol.

[R94] Zakikhani M, Blouin MJ, Piura E, Pollak MN (2010). Metformin and rapamycin have distinct effects on the AKT pathway and proliferation in breast cancer cells. Breast Cancer Res Treat.

[R95] Larsson O, Morita M, Topisirovic I, Alain T, Blouin MJ, Pollak M, Sonenberg N (2012). Distinct perturbation of the translatome by the antidiabetic drug metformin. Proc Natl Acad Sci U S A.

[R96] Suzuki A, Kusakai G, Kishimoto A, Shimojo Y, Ogura T, Lavin MF, Esumi H (2004). IGF-1 phosphorylates AMPK-alpha subunit in ATM-dependent and LKB1-independent manner. Biochem Biophys Res Commun.

[R97] Sun Y, Connors KE, Yang DQ (2007). AICAR induces phosphorylation of AMPK in an ATM-dependent, LKB1-independent manner. Mol Cell Biochem.

[R98] Zajkowicz A, Rusin M (2011). The activation of the p53 pathway by the AMP mimetic AICAR is reduced by inhibitors of the ATM or mTOR kinases. Mech Ageing Dev.

[R99] Ditch S, Paull TT (2012). The ATM protein kinase and cellular redox signaling: beyond the DNA damage response. Trends Biochem Sci.

[R100] Cheema AK, Timofeeva O, Varghese R, Dimtchev A, Shiekh K, Shulaev V, Suy S, Collins S, Ressom H, Jung M, Dritschilo A (2011). Integrated analysis of ATM mediated gene and protein expression impacting cellular metabolism. J Proteome Res.

[R101] Min SH, Goldman ID, Zhao R (2008). Caffeine markedly sensitizes human mesothelioma cell lines to pemetrexed. Cancer Chemother Pharmacol.

[R102] Backus HH, Pinedo HM, Wouters D, Padrón JM, Molders N, van Der Wilt CL, van Groeningen CJ, Jansen G, Peters GJ (2000). Folate depletion increases sensitivity of solid tumor cell lines to 5-fluorouracil and antifolates. Int J Cancer.

[R103] McGuire JJ (2003). Anticancer antifolates: current status and future directions. Curr Pharm Des.

[R104] Gangjee A, Jain HD, Kurup S (2007). Recent advances in classical and non-classical antifolates as antitumor and antiopportunistic infection agents: part I. Anticancer Agents Med Chem.

[R105] Gangjee A, Jain HD, Kurup S (2008). Recent advances in classical and non-classical antifolates as antitumor and antiopportunistic infection agents: Part II. Anticancer Agents Med Chem.

[R106] Denno KM, Sadler TW (1994). Effects of the biguanide class of oral hypoglycemic agents on mouse embryogenesis. Teratology.

[R107] Menendez JA, Cufí S, Oliveras-Ferraros C, Vellon L, Joven J, Vazquez-Martin A (2011). Gerosuppressant metformin: less is more. Aging (Albany NY).

[R108] Cufi S, Corominas-Faja B, Vazquez-Martin A, Oliveras-Ferraros C, Dorca J, Bosch-Barrera J, Martin-Castillo B, Menendez JA (2012). Metformin-induced preferential killing of breast cancer initiating CD44+CD24-/low cells is sufficient to overcome primary resistance to trastuzumab in HER2+ human breast cancer xenografts. Oncotarget.

[R109] Apontes P, Leontieva OV, Demidenko ZN, Li F, Blagosklonny MV (2011). Exploring long-term protection of normal human fibroblasts and epithelial cells from chemotherapy in cell culture. Oncotarget.

[R110] Cole BF (2007). Folic acid for the prevention of colorectal adenomas: A randomized clinical trial. JAMA.

[R111] lrich CM, Potter JD (2007). Folate and cancer--timing is everything. JAMA.

[R112] Xu X, Gammon MD, Wetmur JG, Bradshaw PT, Teitelbaum SL, Neugut AI, Santella RM, Chen J (2008). B-vitamin intake, one-carbon metabolism, and survival in a population-based study of women with breast cancer. Cancer Epidemiol Biomarkers Prev.

[R113] Kerbel RS, Kamen BA (2004). The anti-angiogenic basis of metronomic chemotherapy. Nat Rev Cancer.

[R114] Laquente B, Viñals F, Germà JR (2007). Metronomic chemotherapy: an antiangiogenic scheduling. Clin Transl Oncol.

[R115] Pasquier E, Kavallaris M, André N (2010). Metronomic chemotherapy: new rationale for new directions. Nat Rev Clin Oncol.

[R116] André N, Abed S, Orbach D, Alla CA, Padovani L, Pasquier E, Gentet JC, Verschuur A (2011). Pilot study of a pediatric metronomic 4-drug regimen. Oncotarget.

[R117] Obajimi O, Keen JC, Melera PW (2009). Inhibition of *de novo* purine synthesis in human prostate cells results in ATP depletion, AMPK activation and induces senescence. Prostate.

[R118] Bonafè M, Storci G, Franceschi C (2012). Inflamm-aging of the stem cell niche: breast cancer as a paradigmatic example: breakdown of the multi-shell cytokine network fuels cancer in aged people. Bioessays.

[R119] Hinohara K, Gotoh N (2010). Inflammatory signaling pathways in self-renewing breast cancer stem cells. Curr Opin Pharmacol.

[R120] Lonkar P, Dedon PC (2011). Reactive species and DNA damage in chronic inflammation: reconciling chemical mechanisms and biological fates. Int J Cancer.

[R121] Erol A (2010). Systemic DNA damage response and metabolic syndrome as a premalignant state. Curr Mol Med.

[R122] Vucur M, Roderburg C, Bettermann K, Tacke F, Heikenwalder M, Trautwein C, Luedde T (2010). Mouse models of hepatocarcinogenesis: what can we learn for the prevention of human hepatocellular carcinoma?. Oncotarget.

