# Supplementary material for: Metabolomic fingerprint reveals that metformin impairs one-carbon metabolism in a manner similar to the antifolate class of chemotherapy drugs
Source: Aging (Albany NY). 2012 Jul 22;4(7):480–98. doi: 10.18632/aging.100472 (PMC3433934; doi:10.18632/aging.100472)
Supplement: Supplementary file 1 [file aging-04-480-s001.pdf]

SUPPLEMENTARY FIGURES

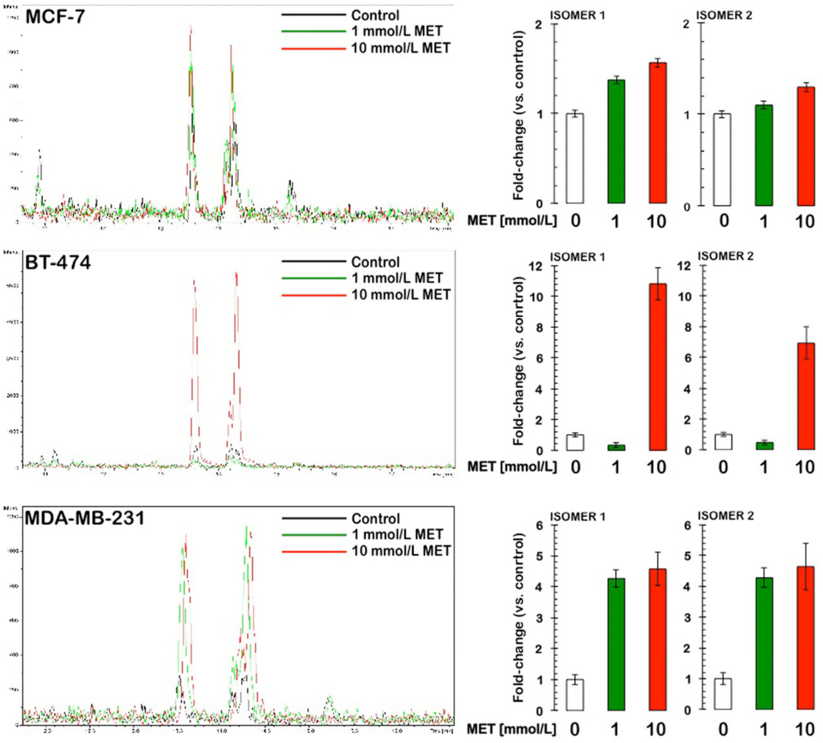

Supplementary Fig. 1. Accumulation of 5-formimino-tetrahydrofolate following treatment with metformin in breast cancer cells. *Left panels.* Figures show enlarged sections of the spectra acquired on aqueous extracts of breast cancer cells following 48 h of treatment with either solvent control, 1 mmol/L metformin or 10 mmol/L metformin, as specified. *Right panels.* Fold-increase in the average content of 5-formimino-tetrahydrofolate following exposure to increasing concentrations of metformin. Values shown are means (columns) ± SD (bars) from three independent experiments.

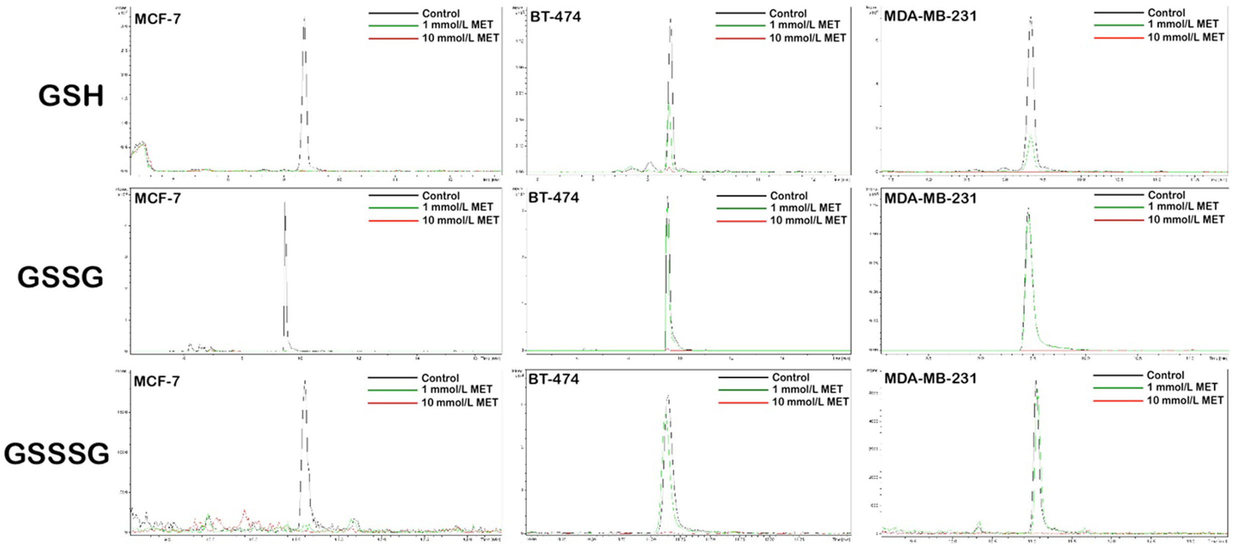

Supplementary Fig. 2. Loss of reduced (GSH), oxidized disulfide (GSSG), and trisulfide (GSSSG) forms of glutathione following treatment with metformin in breast cancer cells. Figure shows enlarged sections of the spectra acquired on aqueous extracts of breast cancer cells following 48 h of treatment with either solvent control, 1 mmol/L metformin or 10 mmol/L metformin, as specified.

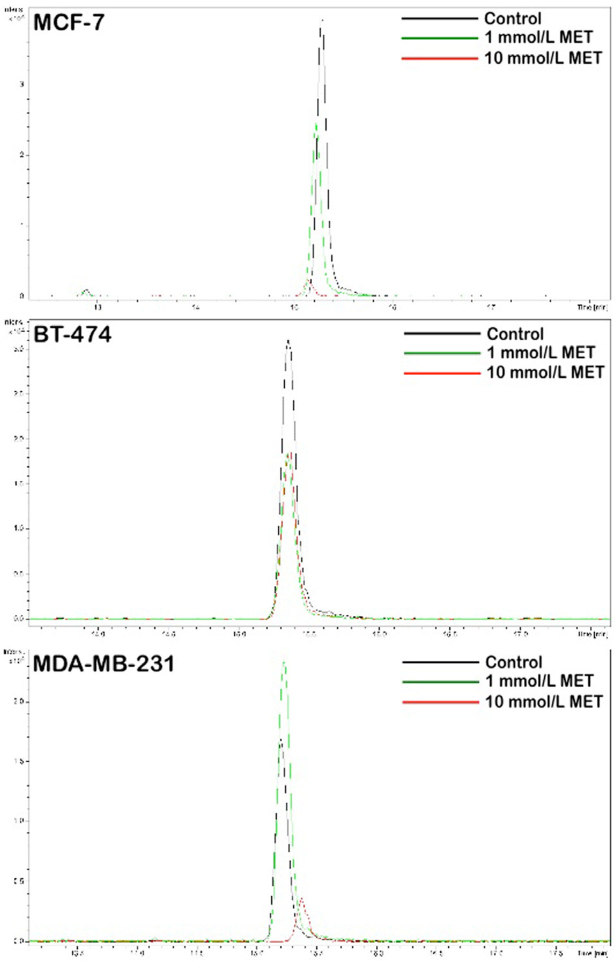

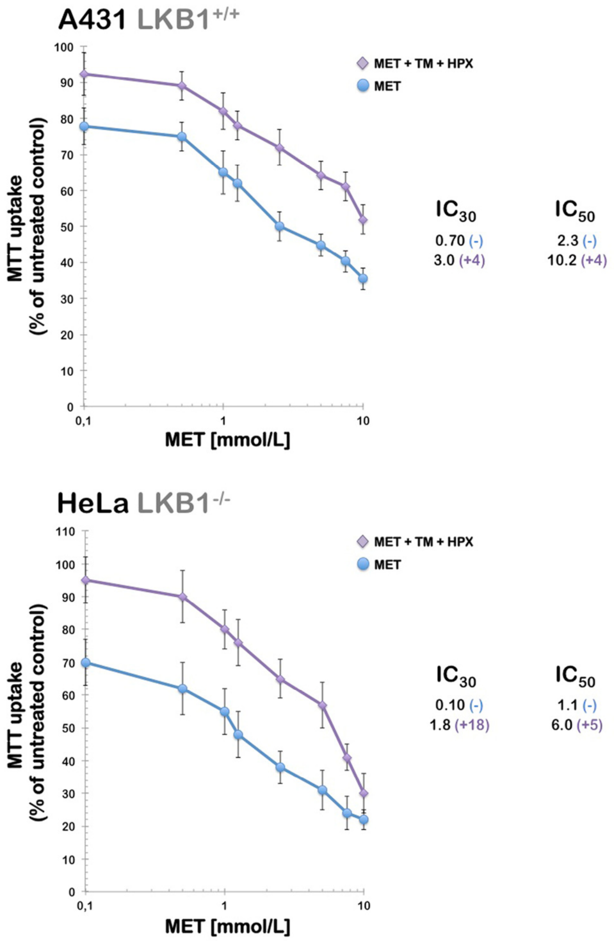

Supplementary Fig. 3. Loss of the amino acid tryptophan following treatment with metformin in breast cancer cells. Figure shows enlarged sections of the spectra acquired on aqueous extracts of breast cancer cells following 48 h of treatment with either solvent control, 1 mmol/L metformin or 10 mmol/L metformin, as specified.

Supplementary Fig. 4. A431 squamous carcinoma cells (top) and HeLa cervical carcinoma cells (bottom) were treated with the indicated concentrations of metformin alone or in the presence of a combination of thymidine (5.6 μmol/L) with hypoxanthine (32 μmol/L). Metformin and modifying agents were added simultaneously and cell growth was determined after 96 h relative to controls without drug processed strictly in parallel using MTT-based cell viability assays. *Right.* The degree of resistance to metformin induced by thymidine and/or hypoxanthine was evaluated by dividing the IC_{30} and IC_{50} values obtained when cells were co-exposed to metformin and pre-formed nucleotides by those obtained in matched control cells cultured in the absence of an exogenous supply of thymidine and/or hypoxanthine. MET, Metformin; TM, Thymidine; HPX, Hypoxanthine.
